# Mining for Microbial Treasure: Recent Therapeutic Leads From Actinobacteria Isolated From the Biodiversity of Australia

**DOI:** 10.1002/cmdc.70569

**Published:** 2026-11-13

**Authors:** Yutong Zou, Nida Ahmed, Constance B. Bailey

**Affiliations:** ^1^ School of Chemistry The University of Sydney Sydney New South Wales Australia

**Keywords:** Actinobacteria, antibiotics, ecology, ecosystems, microbial biodiversity, natural products, secondary metabolites

## Abstract

Australia is one of the most geographically isolated countries in the world, which has resulted in the evolution of a distinct microflora resulting from this unique biodiversity. Actinobacteria are historically one of the most prolific sources of microbial natural products, and the Actinobacteria isolated from Australian soil have led to the discovery of several novel bioactive compounds in recent years. The Australian ecosystem contains diverse rare and novel actinomycetes, including novel *Streptomycetes*, and most remain untapped for new compound discovery. This review summarizes novel natural products and analogs of previously reported compounds which isolated from Actinobacteria collected from isolates gathered across the Australian continent in recent 5 years and discusses the biological activities uncovered. These compounds should be further probed to gain insights about their mode of activity, thus identifying potential leads for next‐generation candidates for anticancer agents, antibiotics, and anthelmintics.

## Graphical abstract

Australia’s geographic isolation has fostered the evolution of a distinctive microbial flora. This review highlights recent discoveries of microbial natural products with therapeutic potential from this unique ecosystem. Further investigation of their mechanisms of action may reveal promising leads for the development of next‐generation anticancer agents, antibiotics, and anthelmintics.

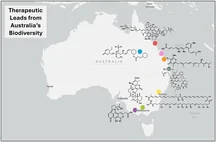

## 1 Introduction

Due to its unique geographic isolation from other landmasses for over 40 million years, Australia harbors remarkable biodiversity [1]. While the public imagination is largely captured by the charismatic megafauna and flora of Australia, the vast lands and aquatic environments are replete with microorganisms that play significant roles in the regulation of the ecosystem [2]. The environmental niches such microorganisms occupy are influenced by various factors, such as pH, nutrients, organic carbon, and nitrogen levels, as well as soil texture. Over the past 20 years, there has been increasing interest in studying the biogeography of these soil microorganisms, including various prokaryotes and eukaryotes such as bacteria, cyanobacteria, microalgae, microfungi, etc. [3]. Nature offers a large repertoire of biologically active substances in the form of secondary metabolites, also called natural products, which are the source of many therapeutic agents. Natural products can be metabolites from plants, fungi, and bacteria, which have been used as conventional medicine since early ages for the treatment of a variety of common diseases and infections due to their evolved bioactivity. The modern healthcare landscape is defined by challenges such as emergence of multidrug resistant (MDR) pathogens that severely compromise efficacy of existing treatment regimens. This is extended to veterinary medicine, where anthelmintic resistance in commercial livestock creates significant food risk and animal welfare [4, 5]. The World Health Organization claims that more than 60%–80% of people rely on traditional medicines to fulfill their healthcare demands [6, 7]. Historically, natural products have been the foundation of widely used pharmaceuticals including penicillin, vancomycin, and the macrolactone avermectins [8]. It is estimated that approximately 50% of the newly developed drugs in the pipeline originate from natural products or their derivatives [1]. Unlike chemical libraries, natural products have exceptional chemical and structural diversity as well as complexity that provides rare chemical scaffolds that are often difficult to replicate by traditional laboratory chemical synthesis methods [9]. This has been a result of evolutionary pressure for millions of years, where microbes adapt in antagonistic and nutrient deficient environments and develop specialized secondary metabolites as chemical defense mechanisms against competitors [10].

Among the producers of these compounds, the phylum Actinobacteria, particularly the genus *Streptomyces*, remains the premier source of bioactive diversity. *Streptomycetes* are ubiquitous aerobic gram‐positive bacteria that grow in both terrestrial and aquatic environments [11]. The development of modern technologies in synthetic biology provides more opportunities for further investigation of natural products. For instance, heterologous expression is a useful tool to activate the “cryptic” or “silent” biosynthetic gene clusters from the original producers. Most of the novel compounds are peptide natural products, and chemical synthesis is difficult due to the structural complexity. Taken together, Australian actinomycetes remain a highly promising reservoir for future drug discovery and development, and they can be further explored utilizing modern synthetic biology technologies. These talented microbes have yielded a wide variety of bioactive classes, which include polyketides [9], peptides [12], alkaloids [13], and macrocyclic spirotetronates [8].

Technological advances are accelerating the pace of these discoveries. Strategies such as microbial diffusion chambers [14] to cultivate fastidious species, miniaturized microbioreactor profiling (MATRIX) [4] for optimized yields, and global natural products social (GNPS) molecular networking [14] for rapid chemical dereplication have accelerated discovery. Additionally, genome mining through algorithms such as AntiSMASH [15] facilitate linking metabolites to their biosynthetic gene clusters (BGCs) as well as predict potential natural products by analyzing the BGC genetic organization.

To highlight the bioactive leads (Table 1) isolated from *Streptomyces* from different habitats across Australia (Figure 1), this review sheds light on the importance of advances in the isolation of previously known as well as novel scaffolds from Australian biodiversity that have been isolated since 2020. These metabolites signify Australia’s enriched environment that enables microbes to produce rare scaffolds that could be used as potential pharmacophores for drug discovery and development for a variety of therapeutics.

**TABLE 1:** Bioactive natural products isolated from *Streptomyces* sp.

Strain	Compound	Activity	Activity type
*Streptomyces* sp. CMB‐M0244 [9]	Mollemycin A	IC_50_ 7 nM (*P. falciparum 3D7*)	Antimalarial
*Streptomyces* sp. MST‐91 080 [16, 17]	Yeppoonic acid B	IC_50_ 2.3 μg/mL (NS‐1 cells)	Anticancer (cytotoxicity)
	Yeppoonic acid C	IC_50_ 3.8 μg/mL (NS‐1 cells)	Anticancer (cytotoxicity)
	Yeppoonic acid A	IC_50_ 50.6 μg/mL (NS‐1 cells)	Anticancer (cytotoxicity)
	Conglobatin B1	IC_50_ 0.084 μg/mL (NS‐1 cells)	Anticancer (cytotoxicity)
	Conglobatin C1	IC_50_ 1.05 μg/mL (NS‐1 cells)	Anticancer (cytotoxicity)
	Conglobatin C2	IC_50_ 0.45 μg/mL (NS‐1 cells)	Anticancer (cytotoxicity)
	Conglobatin D1	IC_50_ 17.8 μg/mL (NS‐1 cells)	Anticancer (cytotoxicity)
	Conglobatin D2	IC_50_ 12.5 μg/mL (NS‐1 cells)	Anticancer (cytotoxicity)
	Conglobatin E	IC_50_ 25 μg/mL (NS‐1 cells)	Anticancer (cytotoxicity)
	Resorculin A	MIC 19.8 μg/mL (*B. subtilis*)	Antibacterial
	Resorculin B	IC_50_ 3.6 μg/mL (NS‐1 cells)	Anticancer (cytotoxicity)
*Streptomyces* sp. CMB‐MW079 [18]	Phoslactomycin E & K	MIC ~ 1 μM (*F. pseudograminearum*)	Antifungal
	Phoslactomycin J	MIC > 12.5 μM (*F. pseudograminearum*)	Antifungal
*Streptomyces* sp. CMB‐PB042 [19]	Glenthenamines B–D, F	Reversal of MDR at 2.5 μM	P‐glycoprotein inhibitor
*Streptomyces* sp. CMB‐PB041 [8]	Glenthmycin A	IC_50_ 1.9 μM (MRSA)	Antibacterial
	Glenthmycin B	IC_50_ 2.2 μM (MRSA)	Antibacterial
	Glenthmycin C	IC_50_ 3.1 μM (*E. faecalis*)	Antibacterial
	Glenthmycin F	IC_50_ 2.9 μM (*VRE*)	Antibacterial
	Glenthmycin H	IC_50_ 2.7 μM (*E. faecalis*)	Antibacterial
	Glenthmycin K 9‐valerate (synthetic derivative)	IC_50_ 0.2–1.6 μM (Gram +ve pathogens)	Antibacterial
*Streptomyces* sp. CMB‐MRB032 [12]	Surugamide A3	EC_50_ 3.3 μg/mL (*D. immitis* mf)	Anthelmintic
	Surugamide AS3	EC_50_ 3.4 μg/mL (*D. immitis* mf)	Anthelmintic
*Streptomyces* sp. MST‐123 921 [20]	Turonicin A	MIC 0.0008 μg/mL (*S. cerevisiae*)	Antifungal
		MIC 0.097 μg/mL (*B. subtilis*)	Antibacterial
*Streptomyces* sp. S4S‐00052A05 [5]	Kendomycin	EC_50_ 4.1 μM (*D. immitis* mf)	Anthelmintic
	Goondomycin A	EC_50_ 0.3 μM (*D. immitis* mf)	Anthelmintic
	Goondomycin B	EC_50_ 3.6 μM (*D. immitis* mf)	Anthelmintic
	Goondomycin C	EC_50_ 2.1 μM (*D. immitis* mf)	Anthelmintic
	Goondomycin D	EC_50_ 21 μM (*D. immitis* mf)	Anthelmintic
	Goondomycin E	EC_50_ 1.3 μM (*D. immitis* mf)	Anthelmintic
	Goondomycin F	EC_50_ 0.5 μM (*D. immitis* mf)	Anthelmintic
	Goondomycin G	EC_50_ 0.5 μM (*D. immitis* mf)	Anthelmintic
*Actinomadura* sp. S4S‐00245B09 [10]	Goondolinone A	EC_50_ 5.1 μM (*D. immitis* mf)	Anthelmintic
	Goondastatin A1	EC_50_ > 13 μg/mL (*D. immitis* mf)	Anthelmintic
	Goondastatin B1	EC_50_ 9.2 μg/mL (*D. immitis* mf)	Anthelmintic
	Goondastatin C1	EC_50_ > 13 μg/mL (*D. immitis* mf)	Anthelmintic
	Goondastatin D1	EC_50_ > 13 μg/mL (*D. immitis* mf)	Anthelmintic
*Streptomyces* sp. S4S‐00061A01 [21]	Goondastatin E1	EC_50_ 4.1 μg/mL (*D. immitis* mf)	Anthelmintic
	Kasichelin C	EC_50_ 0.35 μg/mL (*D. immitis* mf)	Anthelmintic
*Streptomyces* sp. S4S‐00196A10 [22]	Goondapyrone A	EC_50_ 0.05 μM (*D. immitis* mf)	Anthelmintic
	Goondapyrone B	EC_50_ 0.07 μg/mL (*D. immitis* mf)	Anthelmintic
	Goondapyrone C	EC_50_ 0.66 μg/mL (*H. contortus* L1‐L3)	Anthelmintic
	Goondapyrone D	EC_50_ 3.2 μg/mL (*D. immitis* mf)	Anthelmintic
	Goondapyrone E	EC_50_ 2.7 μg/mL (*D. immitis* mf)	Anthelmintic
	Goondapyrone F	EC_50_ 16.7 μg/mL (*D. immitis* mf)	Anthelmintic
	Goondapyrone G	EC_50_ 3.2 μg/mL (*D. immitis* mf)	Anthelmintic
	Goondapyrone H	EC_50_ 3.2 μg/mL (*D. immitis* mf)	Anthelmintic
	Goondapyrone I	EC_50_ 6.5 μg/mL (*D. immitis* mf)	Anthelmintic
	Goondapyrone J	EC_50_ 1.4 μg/mL (*D. immitis* mf)	Anthelmintic
	Actinopyrone A	EC_50_ 0.01 μg/mL (*D. immitis* mf)	Anthelmintic
	Actinopyrone C	EC_50_ 0.02 μg/mL (*D. immitis* mf)	Anthelmintic
*Streptomyces* sp. S4S‐00185A06 [4]	Goondicone A	EC_50_ 21 μM (*D. immitis* mf)	Anthelmintic
	Goondicone B	EC_50_ > 30 μM (*D. immitis* mf)	Anthelmintic
	Goondicone C	EC_50_ > 30 μM (*D. immitis* mf)	Anthelmintic
	Goondicone D	EC_50_ > 30 μM (*D. immitis* mf)	Anthelmintic
	Goondicone E	EC_50_ 10 μM (*D. immitis* mf)	Anthelmintic
	Goondicone F	EC_50_ 11 μM (*D. immitis* mf)	Anthelmintic
	Goondicone G	EC_50_ > 30 μM (*D. immitis* mf)	Anthelmintic
	Goondicone H	EC_50_ > 30 μM (*D. immitis* mf)	Anthelmintic
*Actinomadura* sp. S4S‐00069B10 [23]	Goondicone I + J	EC_50_ 1.1 μM (*D. immitis* mf)	Anthelmintic
	Goondicone K	EC_50_ 13.4 μM (*D. immitis* mf)	Anthelmintic
	Cratermycin A	EC_50_ > 50 μM (*D. immitis* mf)	Anthelmintic
	Cratermycin B	EC_50_ 18.1 μM (*D. immitis* mf)	Anthelmintic
	Cratermycin C	EC_50_ > 50 μM (*D. immitis* mf)	Anthelmintic
	Cratermycin D	EC_50_ > 50 μM (*D. immitis* mf)	Anthelmintic
*Streptomyces* sp. CMB‐STB102 [21]	Goondazine A	EC_50_ 1.2 μg/mL (*H. contortus* L1‐L3)	Anthelmintic
	Goondazine B	EC_50_ 7.2 μg/mL (*D. immitis* mf)	Anthelmintic
*Streptomyces* sp. S4S‐00245A10 [21]	XR334	EC_50_ 6 μg/mL (*D. immitis* mf)	Anthelmintic
	Piperafizine A	EC_50_ > 25 μg/mL (*D. immitis* mf)	Anthelmintic
*Streptomyces* sp. S4S‐00193A39 [24]	Goondoxazole A	EC_50_ 1.38 μM (*D. immitis* mf)	Anthelmintic
	Goondoxazole B	EC_50_ 0.085 μM (*D. immitis* mf)	Anthelmintic
	Goondoxazole C	EC_50_ 0.055 μM (*D. immitis* mf)	Anthelmintic

**FIGURE 1:**
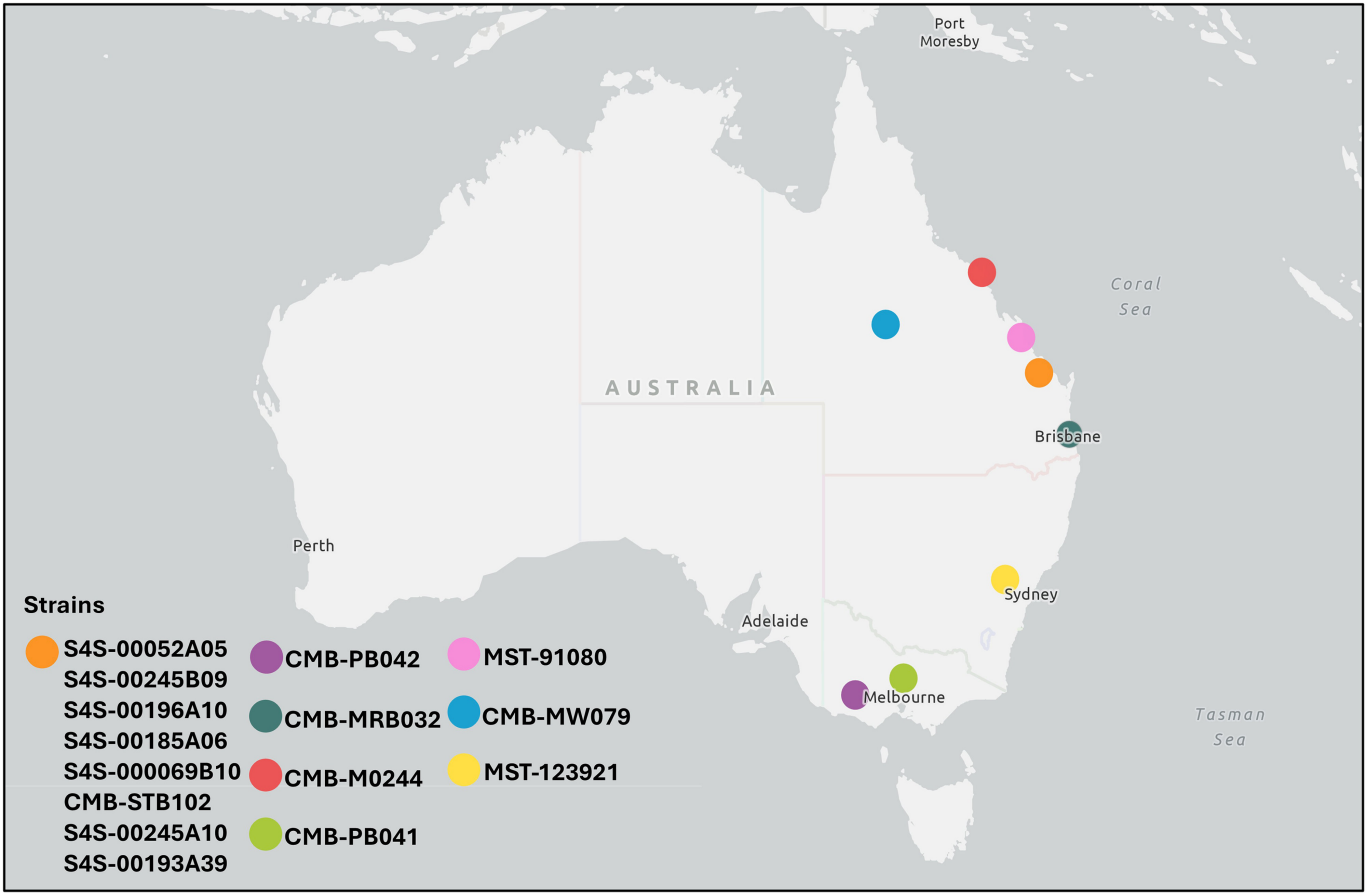
Locations of the strains from which the compounds discussed in this review were isolated (marked as colored dots) collected from the Australian lands.

## 2 Metabolites With Anticancer Activity

The discovery of novel anticancer agents from Australian microbiota highlights the continent’s unique resources for filling the therapeutic gaps in oncology. A handful of leads have been identified which poise new frontiers for anticancer discovery.

### 2.1 Congoblatins

Conglobatins are a family of singly conjugated α, β‐unsaturated macrolide dilactone compounds produced by *Streptomyces*. It was first discovered in 1979 in the *Streptomyces conglobatus* ATCC‐31005 strain [25] known for producing ionomycin [26], which is a polyether antibiotic, during fermentation studies. Recently, new analogs of congloblatins have been discovered in another *Streptomyces* strain, MST‐91080, which is native to Australia [17] and a genetically engineered strain of *Streptomyces pactum* [27]. Typically, the structure of conglobatin consists of two identical monomeric units of 7‐hydroxy‐8‐oxazoyl‐2,4,6‐trimethyl‐2‐octanoic acid joined by two ester linkages. This forms a C_2_ symmetric 16‐membered macrolide architecture that resembles fungal metabolites vermiculin and pyrenophorin [25]. Conglobatins are found in a diverse range of *Streptomyces* species. The 16S rRNA analysis of the Australian strain MST‐91080 that produces six novel analogs (B1, C1, C2, D1, D2, E) validates it as a phylogenetically distinct *Streptomyces* species separate from the original producer [17], indicating either horizontal gene transfer, convergent evolution, or an evolved descendant, highlighting the ecological importance of these compounds. Among the identified molecules, conglobatin B1 exhibits potent cytotoxic activity (IC_50_ of 0.084 μg mL^−1^), based on the biological assay results against NS‐1 mouse myeloma cell line. This showed a 16‐fold increase in activity compared to the parent molecule, which was IC_50_ of 1.39 μg mL^−1^ [17]. Furthermore, the high degree of selectivity for the tumor cell line compared with the normal cells indicates its value as a potential drug candidate.

Conglobatin analogs from MST‐91080 diverge from the parent molecule regarding the methylation pattern on their macrolide skeletons. Conglobatin B1 is a 4′‐desmethyl asymmetric dimer composed of one 2,6‐dimethyl pentaketide and one 2,4,6‐trimethyl pentaketide. C1 is a 4‐4′‐didesmethyl symmetrical analog representing the 2,6‐dimethyl product, while C2 is a 4‐6′‐didesmethyl asymmetric analog that features methyl groups in a varied configuration compared with C1. D1 comprises 4,4′,6′‐tridesmethyl, lacking three methyl groups found in the parent molecule, while D2 is a 2′,4,4′‐tridesmethyl that lacks an olefinic methyl group at the C_2_ position in addition to 2 secondary methyl groups. E is a symmetrical 4,4′,6,6′‐tetradesmethyl analog that has none of the four secondary methyl groups of the parent molecule (Figure 2A) [17]. These variations in the Australian strain point to the polyketide synthase (PKS) may incorporate both malonyl‐CoA and methylmalonyl‐CoA extender units, and the thioesterase (TE) domain dimerizes polyketide variants. The flexibility in the selection of the extender unit and macrolactonization makes them as tractable engineering targets that could plausibly yield tailored methylation patterns on PKS scaffolds that could influence conformational rigidity, membrane permeability, and target binding affinity.

**FIGURE 2:**
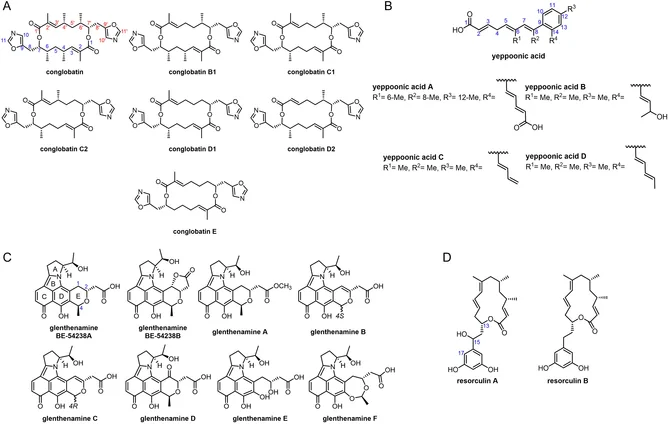
Structures of natural products and their structural analogs with anticancer activity isolated from *Streptomyces* sp. (A) Conglobatins B1, C1, C2, D1, D2, and E. (B) Yepponic acids A‐D. (C) Glentehnamines A‐F, BE‐54238A, and BE‐54238B. (D) Resorculin A and B.

### 2.2 Yeppoonic Acids

Yeppoonic acids are a family of complex bioactive natural products named after their geographic origin. These compounds were isolated from an Australian soil bacterium, *Streptomyces* sp. MST‐91080, from Yappoon, Queensland. These polyunsaturated diene enecarboxylic acid derivatives harbor a 1,2,4‐trisubstituted benzene ring as the central scaffold decorated with specific substitution patterns. Yappoonic acids have structural similarities with closely related 1,2,4‐trisubstituted benzene derivative MF‐EA‐705α/β [28], lorneic acids [29], and NFAT‐133 [30], though it differs in the chain length, methylation pattern, and double bond arrangements. In general, position 1 of yappoonic acids possesses an invariant C_8_ chain containing a styryl diene and enecarboxylate terminus. Position 2 contains C_4_ or C_5_ carbon chain, and position 4 contains a methyl group. Four yeppoonic acid analogs (A–D) are present, each with its unique substitution. Yeppoonic acid A differs at position 2, which has a C_5_ chain originating from the C_14_ chain that terminates in a carboxylic acid group. The yeppoonic acid B exists as a racemic mixture and consists of C_4_ chain at position 2 that terminates in a secondary alcohol. The position 2 of the yeppoonic acid C consists of C_4_ chain that ends in a terminal alkene, whereas the yeppoonic acid D’s position 2 consists of a C_5_ chain that ends at a methyl group (Figure 2B) [16].

Current cancer treatment is available to target rapidly growing cells but has unpleasant side effects often stemming from therapeutics being toxic to both healthy and cancerous cells [31]. Yappoonic acids B and C indicate promising activity and a high degree of selectivity. When tested against NS‐1 mouse myeloma cell line, they showed an IC_50_ of 2.3 ± 0.2 and 3.8 ± 0.8 μg mL^−1^, with moderate activity against the human prostate cancer cell line DU 145, having an IC_50_ of 32.8 ± 2.7 and 49.6 ± 4.1 μg mL^−1^, respectively. However, when tested against nontumor neonatal foreskin fibroblast (NFF) cells, the cytotoxic effects were almost non‐existent even at a concentration of 100 μg ml^−1^, suggesting that these molecules could serve as candidates for further research to develop new therapeutics that could bypass the side effects of chemotherapy.

### 2.3 Glenthenamines

Glenthenamines are complex polycyclic compounds that belong to the class of rare enamine naphthopyranoquinones scaffold having a core framework of five fused rings. They are derived from a type II PKS pathway that undergoes complex transformations, including C‐glycosylation with a deoxyamino sugar precursor. These types of compounds contain both quinone and pyran ring systems in their structure with enamine functionality. These metabolites were first discovered in *Streptomyces* sp. A54238, which was obtained from soil isolates in Japan. The compounds were named BE‐54238A and BE‐54238B and were patented as potential anticancer agents [32]. Later, these were rediscovered from *Streptomyces* sp. MBT76, which was obtained from Chinese soil isolates [33]. Recently, these metabolites were found in *Streptomyces* sp. CMB‐PB042, which originated from a sheep station pasture plant that was collected near Glenthompson, Victoria, Australia [19], yielding four new analogs, i.e., glethenamines B, C, D, and F, and two handling artifacts, i.e., glenthenamaines A and E (Figure 2C). Glenthenamine analogs are distinguished by specific modifications within the pyran ring known as ring E. Diastereoisomers glenthenamine B and C are isomeric didehydro analogs of BE‐54238A that feature a double bond within the ring E. They differ in their configuration, where glenthenamine B possesses a *4S* configuration while glenthenamine C has a 4*R* configuration. Glenthenamine D is the oxidized analog of BE‐54238A, in which the methylene group at the C_1_ position is replaced by a ketone. Glenthenamine E is structurally unique because it possesses the ring A‐D motif but lacks the closed pyran ring E along its associated secondary methyl group. Glenthenamine A is a handling artifact of the parent compound BE‐54238A, where the carboxylic acid moiety at the end of the side chain forms a methyl ester likely due to the exposure of methanol during laboratory processing. Similarly, glenthenamine F, which is an oxidized homolog of the parent compound, is considered a handling artifact formed as the hydrolysis product of glenthenamine E (Figure 2C).

These analogs, along with their parent molecule, have P‐glycoprotein (P‐gp) inhibitory activity, which is involved in MDR [34]. The significance in MDR reversal of glethenamines is notable as A‐D were able to reverse doxorubicin resistance by achieving a gain in sensitivity (GS) of 3.7–5.7 in P‐gp overexpressing human colon carcinoma cells (SW620 Ad300) without having direct cytotoxicity (IC_50_ >30 µM), suggesting them to be potent chemotherapy sensitizers. Furthermore, compared to the clinical standard verapamil with GS 8.1, glenthenamine B, having GS 5.7, achieved approximately 70% efficacy of the positive control with the same concentration [19]. This identified the enamine naphthopyranoquinones scaffold as a novel and promising inhibitory pharmacophore for the development of future MDR reversal agents.

### 2.4 Resorculins

The metabolic profile of *Streptomyces* strain MST‐91080 has yielded several bioactive metabolites, leading to uncovering the resorculins. These are the first ever reported compounds with 3,5‐dihydroxybenzoicacid (DHBA) incorporated into 14 membered macrolide structure, produced by both type I and type III PKS, where resorcinol is used only in larger macrolides as a starter unit [35]. This symmetrical dioxygenated benzene (resorcinol) allows for aromatic stacking and localized hydrogen bonding that can potentially alter the kinetics compared with the other sugar bearing macrolides [36]. Classical macrolide antibiotics (e.g., methymycin, erythomycin, carbomycin) have a 12–16‐membered macrocyclic lactone ring with one or more sugar moieties [37, 38]. The hybrid structure of resorculins differs from the typical glycosylated macrolide motif by fusing a macrocyclic lactone with a phenolic pendant formed by the resorcinol moiety with three methyl groups attached at C_4_, C_6_, and C_8_ [36, 39]. The BGC of resorculins (*rsn*) produces two metabolites, namely resorculin A, which contains a hydroxyl group at C_15,_ and resorculin B, which is a 15‐deoxy analog that has a methylene group at C_15_ (Figure 2D). The dual stream evaluation revealed a distinct spectrum of activity, with resorculin A demonstrating antibacterial activity against *B. subtilis*, while resorculin B exhibited cytotoxic activity against the NS‐1 cell line. This shift in the activity could be attributed to a single structural change at the C_15_ that can significantly alter the biological profile.

## 3 Metabolites With Antimicrobial Activity

Actinomycetes, especially those belonging to the genus *Streptomyces*, are a prime source of chemical diversity with a wide range of biological activity. They have been an abundant source of critical antibacterial classes such as tetracyclines, erythromycins, and rifampicin. The escalating global threat of antimicrobial resistance (AMR) has necessitated a renewed focus on exploring unique and novel therapeutic scaffolds. Recent investigations into the unique biodiversity of Australia including pasture soils, plants, marine sediments, and even insects associated environments like wasp nests have revealed a wealth of complex metabolites that promising to fill gaps in the antibacterial pipeline.

### 3.1 Phoslactomycins

Phoslactomycins are a unique class of polyketides produced by various *Streptomyces* sp. They are characterized by their complex structures that contain an α, β‐unsaturated δ‐lactone, phosphate ester, conjugated diene, and cyclohexane ring [40]. The research on *Streptomyces* sp. CMB‐MW079, isolated from the unique ecological niche of Australian mud dauber wasp nests from Queensland [41], utilizes an integrated discovery workflow of global natural product social (GNPS) molecular networking [42] and MATRIX cultivation [43] to identify a diverse and rare phosphorylated polyketides, including the known phoslactomycin E which was first discovered in *Streptomyces nigrescens* obtained from soil samples in Japan [44], and novel homologs J and K (Figure 3A), alongside unprecedented heterocyclic analogs termed cyclolactomycins and isocyclolactomycins. An unusual defining feature of phoslactomycins is an O_9_ phosphate moiety which makes them rare among the naturally occurring phosphorylated polyketides. Phoslactomycin J contains a methylheptonate group attached at the C_18_ via an ester linkage while phoslactomycin K harbors a cyclohexylpropanoate at C_18_ through ester linkage. Biological evaluation highlights a critical structure–activity relationship (SAR) where the δ‐lactone and C_9_ phosphate motifs are essential for potency; while phoslactomycins E and K demonstrate potent antifungal activity against soil‐borne plant pathogens (MIC ~ 1 µM), the heterocyclic variants that lack these motifs remained biologically inactive [18]. This study validates the importance of exploring untapped environments and employing advanced chemical profiling to expand the known chemical space and functional understanding of the phoslactomycin metabolite class.

**FIGURE 3:**
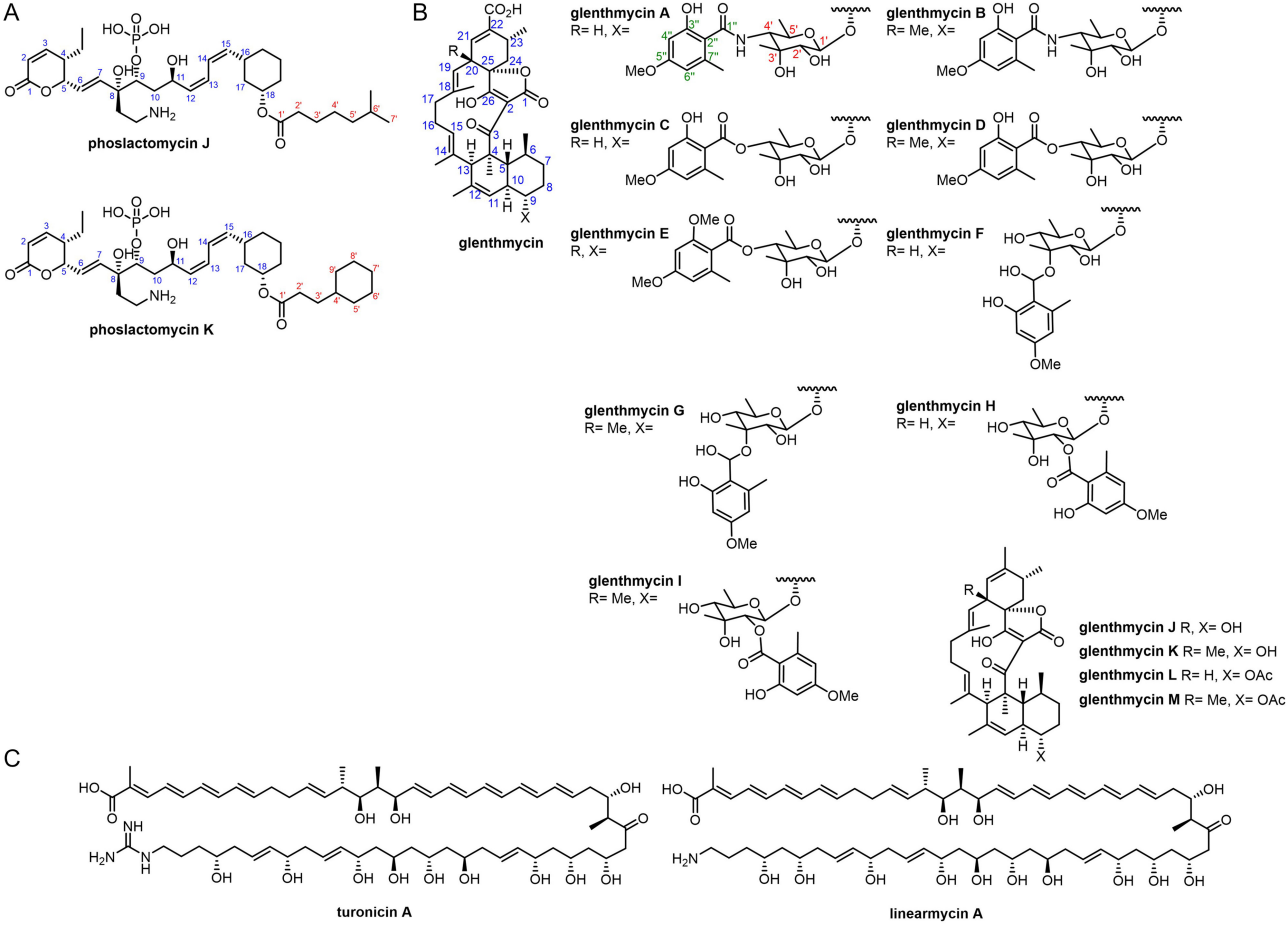
Structures of natural products and their structural analogs with antimicrobial activity isolated from *Streptomyces* sp. (A) Phoslactomycin J and K. (B) Glenthmycin A‐M. (C) Turonicin A and linearmycin A.

### 3.2 Glenthmycins

Glenthmycins are classified as macrocyclic class II spirotetronate scaffolds, which is integral to their bioactivity and pharmacological potential [45]. Isolates of the Australian soil bacterium *Streptomyces* sp. CMB‐PB041 were collected from a sheep pasture plant in Victoria [8]. These polyketide derived metabolites contain a complex moiety consisting of a tetronic acid spiro‐linked to a cyclohexene ring, embedded in a macrocycle that also incorporates a *trans*‐decalin system. This compound was originally isolated as tetromycin C1 from *Streptomyces* sp. MK67‐CF9 reported in a 1996 Japanese patent [46] and was later re‐discovered and renamed to glenthmycin E along with 12 new analogs [8], which resulted in the discrepancy in the literature. In addition, their structural complexity is further enhanced by the presence of unique amino sugar residue, in this case D‐glenthose, and the ability to undergo intramolecular trans‐esterification [8, 47]. Glenthmycin variants A‐M (Figure 3B) have been characterized based on substitutions in the sugar moiety, methylation pattern, regiochemistry of acylation, and functional groups on aglycone core. Glenthmycin A and B are characterized by the presence of d‐glenthose, where glenthmycin B is the homolog of A that features a tertiary methyl group at C_20_. In glenthmycin C‐I, the amino sugar is replaced by a 4‐acylated 6‐deoxyallose moiety featuring a benzoate linkage (O‐acyl). Glenthmycin C is an O‐acyl variant of glenthmycin A, while glenthmycin D is the O‐acyl analog of glenthmycin B. Glenthmycin E is the homolog of glenthmycin D with an additional aromatic methoxy group at the 3″ position. Glenthmycin F‐I are the regioisomers of glenthmycin C and D, where the aromatic O‐acyl substituent is repositioned on the sugar ring. Glenthmycin J‐M are reduced aglycone‐like variants that lack aromatic and sugar substructures on the macrocyclic core. Also, they differ at C_22_ position by having a methyl group instead of a typical carboxylic acid (Figure 3B) [8].

Glenthmycins have antibacterial properties against Gram‐positive bacteria, especially MDR pathogens such as methicillin‐resistant *S.aureus* (MRSA) and vancomycin‐resistant *Enterococci* (VRE). A semisynthetic analog named glenthmycin K 9‐valerate that was derived from glenthmycin K by adding a hydrophobic valerate group to the C_9_ of the macrocyclic core, showed the most potent activity. This diverse suite of metabolites may have evolved as a combinatorial defense mechanism to protect the host organism against microbial competitors in the soil, offering a promising avenue to address critical therapeutic gaps in medicine.

### 3.3 Turonicin A

Turonicin A was first isolated from *Streptomyces* sp. MST‐123921 in the Australia soil sample near the Turon River, and the structure was identified as a linear polyene polyketide [20]. Key examples of them are linearmycins (A‐C), which are identified as antifungal and antibacterial agents [48, 49]. There is also evidence supported that linear polyene polyketide activated signaling involved in bacterial biofilm formation and biofilm morphology [50]. In addition, they can induce natural products biosynthesis in various fungi, and these natural products potentially affect the surrounding microorganisms [51]. Bioinformatic analysis of the MST‐123921 genome provided partial information of the turonicin A BGC but not fully confirmed due to the heavy fragmentation of the remaining parts. Turonicin A has been determined to contain independent pentaene and tetraenone chromophores, which are highly correlated with the structure of linearmycin A (Figure 3C). The current bioinformatic analysis also found seven type I PKS modules that are close homologous to the reported linearmycin BGC, supporting their structural similarity. They have the same chain length, and the only difference is located at the terminus, where turonicin A contains a CH_2_‐CH_2_ group attached to a terminal guanidine, but linearmycin A contains an extra hydroxyl group and has a terminal amine instead. Turonicin A has potent antifungal activities against *Candida albicans* (MIC 0.0008–0.0031 μg/mL) and *Saccharomyces cerevisiae* (MIC 0.0002–0.0008 μg/mL) and moderate antibacterial activities against *Bacillus subtilis* (MIC 0.097–0.39 μg/mL) and *Staphylococcus aureus* (MIC 0.39–3.1 μg/mL). Compared with linearmycin A, turonicin A showed 10‐fold better antibacterial activity and similar antifungal activity. Significantly, the IC_50_ of turonicin A for NFF human fibroblast cell line is larger than 80 µM, indicating low mammalian toxicity [20]. The remarkable antibacterial and antifungal activities and low mammalian toxicity of linear polyene polyketides enable this scaffold to be considered a potential next‐generation candidate for future antibiotic development.

## 4 Metabolites With Anthelmintic Activity

The current agricultural and veterinary anthelmintic products have experienced significant drug resistance, and relative dearth of compounds harboring anthelmintic biological activity makes the resistance easily widespread among them [52]. Thus, novel anthelmintics in novel chemical classes are desperately needed. A recent study collected 30 soil samples from Goondicum Station located near the Burnett River, Queensland, Australia, and isolated 417 chemically unique extracts and isolates. To investigate novel potential anthelmintics, the biological activity screening mainly focuses on *Dirofilaria immitis* microfilariae (mf) motility and *Haemonchus contortus* L1‐L3 larvae. Companion animals such as dogs and cats are easily infected by *D. immitis*, and ruminant livestock such as sheep are more possibly infected by gastrointestinal nematodes such as *H. contortus* [53, 54].

### 4.1 Goondomycins

Carbocyclic ansa‐polyketide goondomycins A‐H and kendomycin were isolated and identified from *Streptomyces* sp. S4S‐00052A05 [5]. The structure and absolute configuration of kendomycin were confirmed in 2020. It contains a quinone methide chromophore and a pentasubstituted tetrahydropyran ring in the macrocycle [55]. The previous literatures have reported several total synthesis routes [56, 57] and biosynthetic strategies [58, 59]. Kendomycin has been reported to have potent antibacterial activity against Gram‐positive and Gram‐negative bacteria, including *S. aureus* (MRSA) strains (e.g., MIC for STA and MU50 recorded as 3.9 and 7.8 μM, respectively). It shows remarkable cytotoxic activity against three different tumor cell lines, including stomach adenocarcinoma, hepatocellular carcinoma, and breast adenocarcinoma [55]. The recent study examined antiparasitic activity of kendomycin and determined it had moderate activity against *D. immitis* mf motility inhibition (EC_50_ 4.1 µM). Goondomycins A‐H were identified as the new kendomycin‐like carbocyclic ansa‐polyketides. Goondomycins A‐B, G‐H had the same quinone methide chromophore with kendomycin, while goondomycins C‐F contained new chromophores (Figure 4A). Goondomycins A‐G have no antibacterial activity against *E. coli* and *C. albicans* and only moderate activity against *S. aureus*. Only goondomycin B showed moderate cytotoxic activity against human colorectal and lung carcinoma cells. Goondomycins A‐G showed better activity against *D. immitis* mf motility inhibition compared to *D. immitis* L4 and *H. contortus* L1‐L3 larvae. Among them, goondomycins A and F had a potent activity against *D. immitis* mf motility (EC_50_ 0.3 and 0.5 µM, respectively) and less extent activity against *D. immitis* L4 larvae (EC_50_ = 1.4 and 1.8 μM, respectively) [5]. This activity profile against both microfilaria and L4 larvae is attractive, since activity for infective (mf) and developing (L4) is important for prophylactic veternary therapy against this parasite in dogs and cats.

**FIGURE 4:**
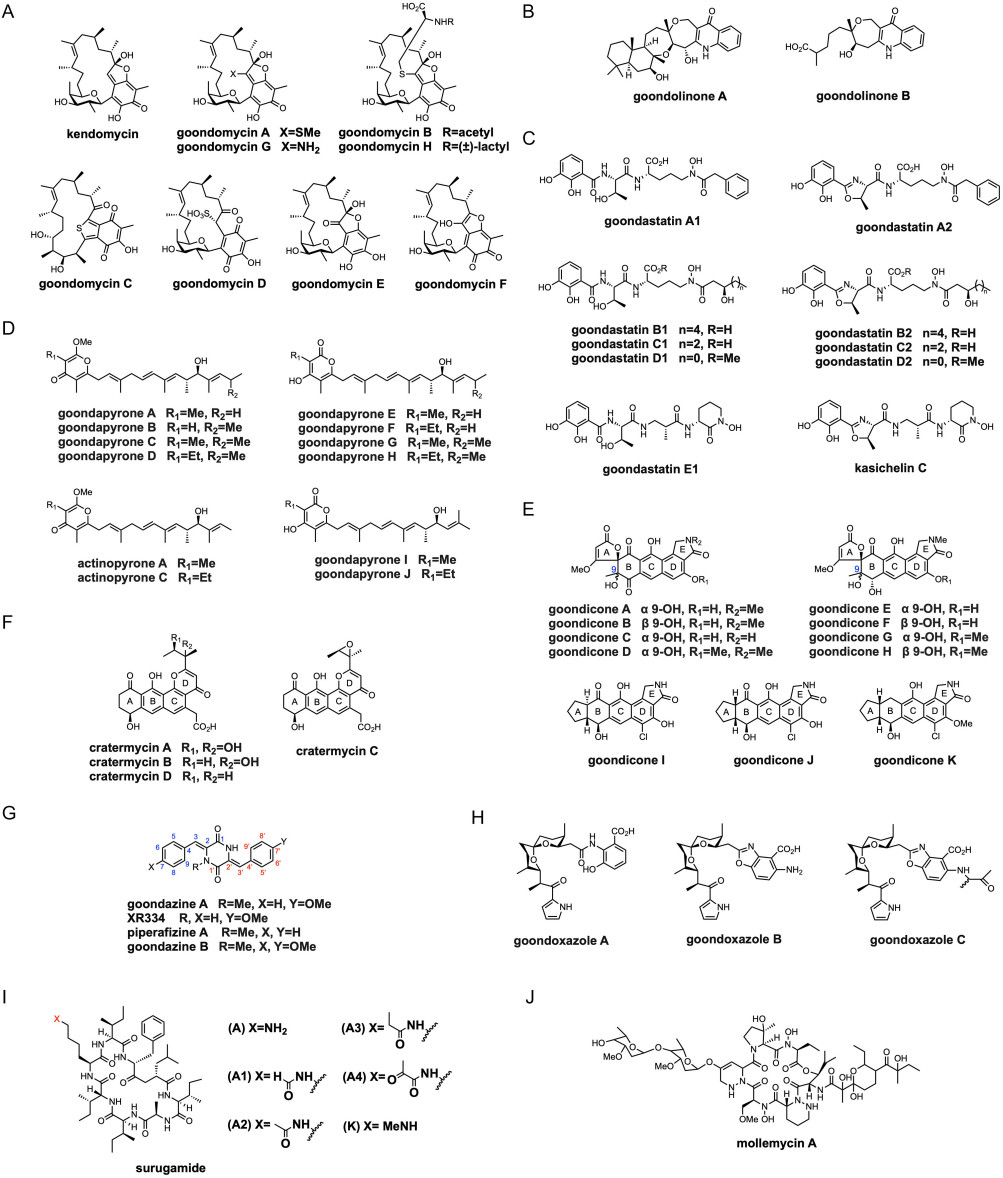
Structures of natural products and their structural analogs with anthelmintic activity isolated from actinomycetes. (A) Kendomycin and Goondomycin A‐H. (B) Goondolinone A and B. (C) Goondastatin A1‐E1, A2‐D2, and Kasichelin C. (D) Goondapyrone A‐J, Actinopyrone A and C. (E) Goondicone A‐K. (F) Cratermycin A‐D. (G) Goondazine A and B, XR334, and piperafizine A. (H) Goondoxazole A‐C. (I) Surugamide. (J) Mollemycin A.

### 4.2 Goondolinones and Goondastatins

Goondolinones A and B were discovered from *Actinomadura* sp. S4S‐00245B09 that derived from the samples collected from Goondicum Station in Queensland. The structures of goondolinones A and B were identified as unprecedented terpenyl‐quinolin‐4(1H)‐ones. They are the first examples of prenylated quinoline‐4(1H)‐one that fuse carbo/heterocyclic terpenyl substructures with quinoline‐4(1H)‐one. In particular, goondolinone A consists of two cyclohexyl, two 7‐membered oxepanes, and quinoline‐4(1H)‐one chromophore, while goondolinone B contains a terminal C‐1 carboxylic acid, and the 7‐membered cyclic ether fused to the quinoline‐4(1H)‐one (Figure 4B) [10]. Quinoline‐4(1H)‐ones have been confirmed with a broad range of biological activities and applied in therapeutics [60]. Intervenolin was isolated from *Nocardia* sp. ML96‐86F2 and showed potent anti‐tumor activity against human gastric and colorectal cancers [61]. Other known examples include aurachins family which are 3‐farnesyl‐4(1H)‐quinolone. Aurachins A‐D were first isolated from *Stigmatella aurantiaca* strain Sg a15 and other members E‐R were identified later. Aurachins C and D showed excellent antimicrobial activity than A and B, and aurachin D also has potent antimalarial activity [62]. Goondolinones A and B were proposed to be biosynthesized from the prenylation of geranyl–geranyl or geranyl with a quinoline‐4(1H)‐one precursor, respectively, followed by oxidation and cyclization. Goondolinone A only showed moderate motility inhibition of both *D. immitis* microfilaria and L4 larvae (EC_50_ 5.1 and 21.4 µM, respectively [10]. However, its novel structure class and anthelmintic mechanism may not be affected by current resistance pathways, therefore inspiring the development of new anthelmintics.

Further investigation of *Actinomadura* sp. S4S‐00245B09 found that the strain also produced the second class of natural products which were identified as new triamide‐hydroxamates, goondastatins A1‐D1. Analysis of the strain extracts also detected their corresponding oxazoles, goondastatins A2‐D2. Additionally, goondastatin E1 and the known oxazole kasichelin C were isolated from *Streptomyces* sp. S4S‐00061A01. Kasichelin C was previously isolated from Kilimanjaro soil‐derived *Actinomyces* sp. K17/9 [63]. Goondastatins A1‐D1 were confirmed to have the 3,4‐dihydroxybenzamide (DHB) moiety, L‐Thr, and 5‐N‐OH‐Orn residues but differed in the terminal moiety. Goondastatin A1 contained phenylacetic acid, while goondastatins B1‐D1 contained 3‐hydroxy fatty acid with different chain lengths. Goondastatin E1 featured the same 3,4‐dihydroxybenzamide (DHB) moiety, L‐Thr, but 2‐methyl‐β‐d‐Ala replaced the 5‐N‐OH‐Orn moiety and connected to a 5‐N‐OH‐D‐Orn lactam ring (Figure 4C). Goondastatins A1‐E1 are NRPS tripeptides, and the DHB‐Thr moiety is known as a catecholate siderophore structural feature [21]. Siderophores are produced by various microorganisms, which can form a stable complex with ferric iron, to regulate cellular iron availability [64, 65]. Recently, siderophores were applied as a useful tool to precisely transport antibiotics into the cell [66]. The analysis of goondastatins A1‐E1 structure was consistent with oxazole ring‐opened hydrolysis transformation of their corresponding oxazoles goondastatins A2‐D2 and kasichelin C (Figure 4C). These corresponding oxazoles were also detected in previous known examples which share the same DHB‐Thr moiety, including agrobactin [67] and serratiochelin [68]. Goondastatin B1 had moderate activity against *D. immitis* mf motility (EC_50_ 9.2 μg/mL), but goondolinone A isolated from the same strain had a better inhibition (EC_50_ 2.5 μg/mL) [10]. Kasichelin C exhibited a potent inhibition of the *D. immitis* mf motility (EC_50_ 0.35 μg/mL), which is the most effective one in the goondastatins family.

The two different classes detected in the same strain possibly indicate that multiple BGCs exist; thus, bioinformatic analysis and heterologous expression are worthwhile to apply to identify which BGC encodes which secondary metabolite. The minor cometabolite oxazoles of goondastatins may have different BGCs from goondastatins or have modification enzymes encoded. Further insight into biosynthetic mechanisms is essential for potential enzyme engineering and future pathway optimization.

### 4.3 Goondapyrones

New compounds goondapyrones A‐J and known compounds actinopyrones A and C were identified from the extract of *Streptomyces* sp. S4S‐00196A10 [22]. Actinopyrones A and C were first isolated by Yano et al. in 1986 and were identified in their γ‐pyrone scaffold structure [69]. Goondapyrones A‐D were confirmed to have the same γ‐pyrone scaffold (Figure 4D). γ‐Pyrone natural products have been found in various organisms and have shown pharmacological activity in antibacterial, antifungal, and anticancer [70, 71]. In contrast, Goondapyrones E‐J contain an α‐pyrone structure (Figure 4D). α‐Pyrone is also known to exhibit a wide range of biological activities, including HIV protease inhibiting and telomerase inhibiting effects [72]. Yano et al. reported that actinopyrones A and C have coronary vasodilating activity, though actinopyrone C showed 10‐fold less potency compared with actinopyrone A. Additionally, only limited microbial activity against some Gram‐positive bacteria was demonstrated, but no activity against Gram‐negative bacteria and yeasts [73]. Actinopyrone A was later reported to have a potent anti‐*Helicobacter pylori* activity with a MIC value of 0.0001 μg/mL [74], and moderate antifungal activity against *Ganoderma boninense* [75]. Goondapyrones A‐B, actinopyrones A and C showed potent inhibition against the motility of *D. immitis* microfilaria (EC_50_ 0.01–0.07 μM) but very limited activity against the L4 larvae. These four with goondapyrones C also showed potent inhibition activity against *H. contortus* L1‐L3 larvae development (EC_50_ 0.15–0.66 μM) [22]. The anthelmintic activity of goondapyrones A‐B, actinopyrones A and C was 1000‐fold greater than goondapyrones E‐J, supporting the structure activity relationship that the γ‐pyrone scaffold is more effective than the α‐pyrone scaffold. The potent activity of goondapyrones A‐B, actinopyrones A and C against both *D. immitis* microfilaria and *H. contortus* L1‐L3 larvae indicates their potential wide application as anthelmintics.

### 4.4 Goondicones

Goondicones A‐H were first isolated from the *Streptomyces* sp. S4S‐00185A06, while goondicones I‐K were found in *Actinomadura* sp. S4S‐000069B10 later [4, 23]. The structure of goondicones A‐H was identified as a rare class of spiro‐isoindolinones. They are pentacyclic compounds, and ring A is a spiro moiety; specifically, goondicones E‐H contain the reduced hydroxyl on ring B instead of the carbonyl in goondicones A‐D (Figure 4E) [4]. However, goondicones I‐K were identified as isoindolinones, which featured an unprecedented carbo‐heterocyclic framework. They also contained five rings and a cyclopentane ring A moiety fused to ring B, and they all had a chloro group on ring D (Figure 4E) [23]. Isoindolinones scaffold has widely existed in various natural products and possesses biological activities including antimicrobial, analgesic, anti‐inflammatory, and anticancer [76, 77, 78, 79]. The isoindolinone skeleton has also demonstrated significant potential in agrochemical development due to activity in insecticidal, herbicidal, and plant growth regulation [80]. Spiro‐isoindolinones are derived from isoindolinone and have been found in bioactive natural products and pharmaceutical candidates [81]. The structures of goondicones A‐H were characterized to be in the same class as lactonamycin and lactonamycin Z. Lactonamycin was reported to have potent antimicrobial activity against Gram‐positive bacteria and cytotoxicity against various tumor cell lines, while lactonamycin Z showed moderate antimicrobial activity and only potent activity against gastric adenocarcinoma (HMO2) [82, 83]. Based on the structural features of goondicones A‐K, they are proposed to be biosynthesized by an NRPS‐PKS pathway, starting with a glycine unit connected to nine acetate units [4].

Goondicones A‐H did not inhibit the motility of *H. contortus* L1‐L3 larvae and only goondicones A, E and H showed moderate activity against *D. immitis mf*, with EC_50_ from 10–21 μM [4]. By contrast, the equilibrating mixture goondicones I + J exhibited potent antimicrobial activity against *S. aureus* ATCC 25923 (IC_50_ 0.13 μM), also cytotoxicity against human colorectal carcinoma cells (SW620) (IC_50_ 0.26 μM) and lung carcinoma cells (NCI‐H460) (IC_50_ 0.11 μM). Although the same as goondicones A‐H, I‐K did not inhibit the motility of *H. contortus* L1‐L3 larvae, the equilibrating mixture of goondicones I + J was 10‐fold more potent against *D. immitis mf* (EC_50_ 1.1 μM) than the previously reported strongest goondicone E (EC_50_ 10 μM), along with similar inhibitory activity to *D. immitis* L4 larvae motility [4, 23].

This structure–activity relationship between goondicones A‐H and I‐K is noteworthy to further investigate, providing additional insights into the goondicone anthelmintic pharmacophore. Additionally, the identification of BGCs of goondicones A‐H and I‐K will enable us to understand the biosynthetic mechanisms and different modifications involved. Heterologous expression may also provide the opportunity to obtain individually stable goondicone I and J, which can be more effectively screened for biological activity.

### 4.5 Cratermycins

The cultivation of *Actinomadura* sp. S4S‐000069B10 also revealed new anthra‐γ‐pyrone cratermycins A‐D [23]. γ‐Pyrone based natural products are widely found in biologically active metabolites and considered a valuable scaffold in medicinal chemistry libraries [70, 71]. The natural aromatic ring fused γ‐pyrone belongs to a class of polyketides, which possess various biological activities and are applied in the pharmaceutical and cosmetic industry [84, 85]. Cratermycins A‐D feature the same skeleton, which contains a cyclohexyl fused with the naphthalene and γ‐pyrone ring. Different from the other three, cratermycin C has an epoxide substituted on the γ‐pyrone ring (Figure 4F). Their biosynthetic route was proposed to start from the compound synthesized by PKS conjugation of ten acetates and one propionate [23]. The structure of cratermycins A‐D is highly similar to the known examples in the anthra‐γ‐pyrone class: kapurimycins A1‐A3 isolated from Japanese soil‐derived *Streptomyces* sp. DO‐115 [86] and shellmycins A‐D isolated from the Chinese marine shell sediment‐derived *Streptomyces* sp. shell‐016 [87]. They all contain the same fused ring system and a C‐1 carboxylic acid substituted on the ring C. Kapurimycin A3 was shown to have potent antimicrobial, antitumor, and cytotoxic activity [88], and shellmycins A, B, and D had potent anticancer activity. However, cratermycins did not exhibit any antimicrobial, antifungal, or anticancer activity, and only cratermycin B exhibited moderate activity against *D. immitis* mf motility with an EC_50_ of 18.1 μM.

### 4.6 Goondazines

Goondazines A and B were isolated from *Streptomyces* sp. CMB‐STB102 and reported as new piperazines. The cultivation of *Streptomyces* sp. S4S‐00245A10 also found the new piperazines, goondazine A and known piperazines, XR334 and piperafizine A [21]. Goondazine A was found as an isomer of the known piperazine XR330, which was isolated from the same strain as XR334. XR334 and XR330 were previously isolated from UK soil‐derived *Streptomyces* sp. XO1/4/100 and reported as plasminogen activator inhibitor‐1 antagonists [89], and XR334 was later reported as an antimitotic agent [90]. Piperafizine A was previously found in Fiji soil‐derived *Streptoverticillium aspergilloides* Q576‐2 and was shown as a vincristine cytotoxicity potentiator [91]. Goondazine A, B, XR334, and piperafizine A have the same scaffold 2,5‐diketopiperazine (2,5‐DKP) formed by the condensation of phenylalanine and tyrosine and contain unsaturated C‐C double bonds at 2 and 2′ positions of the 2,5‐DKP ring (Figure 4G). 2,5‐DKP is the smallest cyclodipeptide and widely exists in natural products. Their conformationally constrained heterocycle provides better structural rigidity and higher stability to proteolysis, making this class attractive in drug discovery [90]. They have been reported to have antimicrobial, anticancer, antioxidation, and anti‐Alzheimer activity [92, 93]. Goondazine A had moderate activity against *D. immitis* mf motility (EC_50_ 15 μg/mL), while it inhibited *H. contortus* L1‐L3 larval development to a larger extent (EC_50_ 1.2 μg/mL). This potent and selective activity against *H. contortus* L1‐L3 larvae can be further applied in the development of anthelmintics for ruminant livestock, including sheep and goats, which will improve productivity and reduce their mortality.

### 4.7 Goondoxazoles

Goondoxazoles A‐C were discovered as new examples of pyrrolo‐spiroketal polyketide from *Streptomyces* sp. S4S‐00193A39. The structure of goondoxazole A was identified as containing an α‐ketopyrrole, a spiroketal ring, and a disubstituted benzoic acid moiety, while goondoxazoles B and C contained a substituted benzoxazole instead of the benzoic acid ring. Goondoxazoles B and C differed in the substitution in the benzoxazole moiety. Specifically, goondoxazole B had an amino group, and the one on goondoxazole C was N‐alkylated with a 3‐amino‐2‐butanone (Figure 4H) [24]. These structural features of goondoxazoles are classified as a specific PKS‐NRPS‐derived natural products and are well‐known by their first example, calcimycin (A23187). Calcimycin was first reported as a polyether divalent cation ionophore secondary metabolite and isolated from *Streptomyces chartreusis* NRRL 3882 [94, 95]. It has been shown that calcimycin acts as a Ca^2+^ ionophore, activates the intracellular signal, and induces apoptosis [96]. Calcimycin has also been used to understand the calcium concentration effect in heart disease, high blood pressure, and brain disease [97, 98, 99]. Calcimycin family also includes cezomycin isolated from the same strain as calcimycin [100] and precezomycin isolated from *Kitasatospora putterlickiae* 10–13 [101]. Goondoxazoles A‐C exhibited potent inhibition against *D. immitis* mf and *D. immitis* L4 larvae motility, while goondoxazoles B, C, and calcimycin (EC_50_ 0.027–0.085 μM) were more potent against *D. immitis* mf than goondoxazole A (EC_50_ 1.38 μM). This observation showed that the benzoxazole moiety affects the activity. Therefore, they further investigated other benzoxazoles isolated from Goondicum pasture soil‐derived strains. Among them, the known benzoxazole A‐33583 which was isolated from *Streptomyces* sp. S4S‐00200B03 showed comparable activity against *D. immitis* mf (EC_50_ 0.072 μM) and five‐fold more potency against *D. immitis* L4 larvae (EC_50_ 0.43 µM). This evidence demonstrates that benzoxazoles have potent and selective activity against *D. immitis*, and it can be replicated and even improved by using simpler analogs.

### 4.8 Surugamides

Surugamides are a relatively rare class of cyclic octapeptides produced by taxonomically diverse *Streptomyces* species found in both terrestrial and marine sediments. Surugamide A‐E were first isolated from marine *Streptomyces* near the coast of Japan [102]. Later, a new N‐methylated analog was discovered along with previously known surugamide A from *Streptomyces* sp. CMB‐MRB032 isolated from sheep fecal sample collected from Bathurst, Victoria, Australia. These metabolites, including surugamide A that incorporates a d‐allo‐Ile2 residue rather than d‐Ile2 and the novel N‐methylated analog surugamide K have recently gained attention for their potential as selective anthelmintics (Figure 4I) [12]. Biological screening revealed that acyl‐surugamides such as surugamide A3 and semi synthetic AS3 as potent inhibitors of heartworm (*D. immitis*) motility, showing EC_50_ between 3.3. and 3.4 µg/mL. Extensive structure activity relationship studies indicated that the selective acylation of Lys3‐Ɛ‐NH_2_ is critical for achieving their anthelmintic effect. Unlike highly toxic antimycins that are often co‐produced by these strains, surugamides exhibit no significant cytotoxicity toward mammalian cells and lack general antibacterial and antifungal activity. The novel targeted mode of action is proposed to the effect of specific binding and/or modification of a specific molecular target of the parasite instead of cell wall disruption, suggesting a target‐specific mode of action for these compounds.

### 4.9 Mollemycin A

Mollemycin is a glyco‐hexadepsipeptide‐polyketide belonging to the azinothricin family, which was first discovered and isolated from a marine‐derived sediment sample of Australian *Streptomyces* CMB‐M0244, collected (−55 m) off South Molle Island, Queensland, Australia [103]. This hybrid structure consists of three biosynthetic elements, such as a cyclic hexapeptide that is attached to a polyketide residue at the (2S, 3S)‐3‐hydroxyleucine and a disaccharide moiety at the γ‐oxopiperazic acid (Figure 4J). It is the only azinotricin compound to be reported that is glycosylated with two L‐oleandrose units, which has been known to be a functional contributor in antiparasitic activity [104]. This compound produces a promising drug candidate due to minimal cytotoxic effects on mammalian cells and remarkable potency in growth inhibition for gram‐positive and negative bacteria, as well as MDR and drug‐sensitive malarial strains in vitro [105]. The BGC comprises PKSs/NRPSs hybrid that encodes for one type I PKS module and six NRPS modules. In addition to this, several other genes encode for disaccharide sugar (*momZ2‐8*), glycosyl‐transferase (*momZ1*), LmbU‐like, and AfsR/SARP transcriptional regulator [9]. Compared with conventional PKSs that typically employ acetyl‐CoA and malonyl‐CoA as the starter unit to make C_2_ or C_3_ chains [106], mollemycin uses 2‐methylbutylmalonyl‐CoA, a seven carbon unit, suggesting the PKS module of mollemycin is capable of accepting wider range of substrates, implicating the ability to accommodate bulkier and structurally diverse extender units. The enzyme machinery that carries out substrate selection with broader active site tolerance than canonical acetyl‐CoA/malonyl‐CoA modules can be reprogrammed to generate novel polyketides with altered carbon skeletons, chain length, or branching patterns.

## 5 Conclusion

Australia harbors diverse actinomyces due to its unique terrestrial and marine environments, and most of them remain uncharacterized. Over the last few decades, researchers have been interested in investigating the untapped Australian ecosystem. Novel compounds have been reported to exhibit potent biological activity and an unprecedented scaffold. In this review, we focus on novel compounds and analogs of previously reported compounds which isolated from Australian actinomyces after 2020, and their structural features and biological activities. Among them, K 9‐valerate and turonicin A have been demonstrated to possess potent antibacterial activity against multiple Gram‐positive bacteria and Gram‐negative bacteria [8, 20]. This highly supported the unexplored potential of secondary metabolites in Australian actinomyces and the potential to address threatening drug resistance. Additionally, several novel compounds isolated from Australian actinomyces have been found to have a highly similar structure to previous compounds isolated from other regions. For instance, cratermycins and goondoxazoles found in Goondicum Pastoral, Queensland, showed high similarity in structure with shellmycins isolated from Binzhou shell island, China, and precezomycin isolated from the Ryukyu islands, Japan, respectively. This significant structural similarity between compounds isolated from distinct geographical origins may reflect that they shared evolutionary ancestry; however, the confirmed assumption requires the genome and BGCs comparison of these Australian strains and the previously reported producer [107]. *Streptomyces* have been well developed as a host organism that is suitable to express biosynthetic gene clusters from actinomyces [108]. Therefore, heterologous expression is a valuable strategy for scaleup, and sufficient quantities enable more biological activity screening. Recently, cell free expression strategy has been largely applied to complex natural products. *Streptomyces* cell free expression platform has been established using different strains and has synthesized type III and type I polyketide synthases [109]. There have been reports that cell free extract provides a high‐throughput approach for antimicrobial discovery, which discovered a dipeptide antibiotic TAN1057 A/B (GS7128) and a nonribosomal peptide GE81112 [110, 111]. Thus, such novel approaches can be widely applied in antimicrobial discovery and the study of antimicrobial resistance and potentially be further used in mimicking cell biochemistry and probing structure activity relationships.

## Funding

This study was supported by The University of Sydney School of Chemistry, The University of Sydney Infectious Diseases Institute, The University of Sydney Centre for Drug Discovery Innovation, and The Sydney Anti‐Biotics Accelerator.

## Conflicts of Interest

The authors declare no conflicts of interest.

## Data Availability

Data sharing not applicable to this article as no datasets were generated or analyzed during the current study.
